# The association of the medial meniscus covering ratio with knee cartilage thickness at 15 medial compartment subregions as found by three‐dimensional analysis using MRI

**DOI:** 10.1002/jeo2.70005

**Published:** 2024-08-27

**Authors:** Nobutake Ozeki, Hideyuki Koga, Tomomasa Nakamura, Hiroki Katagiri, Yusuke Nakagawa, Takashi Hoshino, Mai Katakura, Masaki Amemiya, Aritoshi Yoshihara, Hisako Katano, Mitsuru Mizuno, Kentaro Endo, Jun Masumoto, Ichiro Sekiya

**Affiliations:** ^1^ Center for Stem Cell and Regenerative Medicine Tokyo Medical and Dental University Tokyo Japan; ^2^ Department of Joint Surgery and Sports Medicine, Graduate School of Medical and Dental Sciences Tokyo Medical and Dental University Tokyo Japan; ^3^ Department of Cartilage Regeneration Tokyo Medical and Dental University Tokyo Japan; ^4^ Clinical Center for Sports Medicine Tokyo Medical and Dental University Tokyo Japan; ^5^ Fujifilm Corporation Tokyo Japan

**Keywords:** cartilage thickness, knee, meniscus coverage ratio, meniscus extrusion, MRI 3D analysis

## Abstract

**Purpose:**

The correlation of cartilage thickness measured by three‐dimensional (3D) magnetic resonance imaging (MRI) and the medial meniscal coverage ratio (MMCR), which presented pathology of the medial meniscus extrusion (MME) in 3D MRI, has not yet been elucidated. The study's purpose was to retrospectively verify whether the average cartilage thickness calculated by the automatic MRI 3D analysis system for each subregion was correlated with MMCR.

**Methods:**

A total of 60 patients underwent medial meniscus repair or high tibial osteotomy to treat their medial knee osteoarthritis. Cartilage thickness and MMCR were automatically calculated using 3D MRI software. The MMCR was defined as the ratio of the area covered by the meniscus within the medial tibial cartilage area to the total medial tibial cartilage area. The association between MMCR and the average cartilage thickness at 15 subregions in the medial femoral condyle (MFC) and medial tibial plateau (MTP) was evaluated using Spearman's rank correlation coefficient.

**Results:**

Kellgren–Lawrence grade exhibited a negative correlation with MMCR and a positive correlation with MME width. Cartilage thickness in the MTP had a moderately positive correlation with MMCR at four subregions and a weakly positive correlation at another subregion. Cartilage thickness in the MFC showed a moderately positive correlation with MMCR at five subregions and a weakly positive correlation at one subregion.

**Conclusions:**

Cartilage thickness calculated by automatic MRI 3D analysis system had a positive correlation with MMCR for all subregions of the anterior and middle subregions in the MFC and for five regions of nine subregions of the anterior and middle subregions in the MTP.

**Level of evidence:**

Level II, cross‐sectional study (diagnosis).

## Graphical abstract

Cartilage thickness calculated by automatic three‐dimensional magnetic resonance imaging analysis system had a positive correlation with medial meniscus covering ratio for all subregions of the anterior and middle subregions in the medial femoral condyle and for five regions of nine subregions of the anterior and middle subregions in the medial tibial plateau.

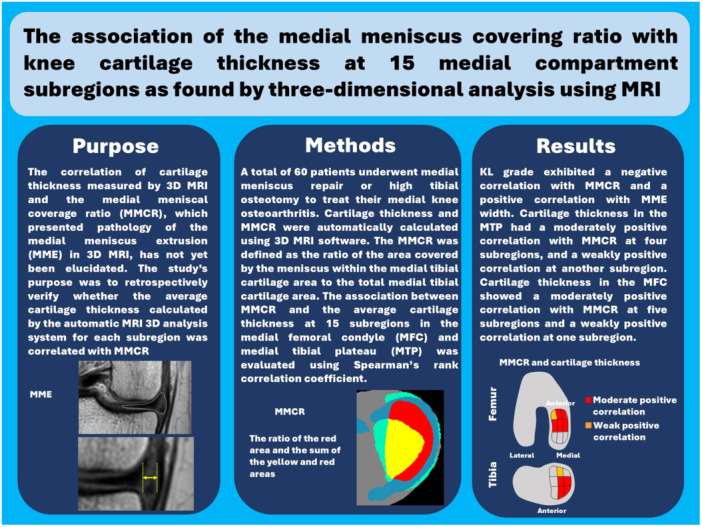

**Abbreviations**
3D MRI: three‐dimensional MRI
acMF: anterior central MF
acMT: anterior central MT
aeMF: anterior external MF
aeMT: anterior external MT
aiMF: anterior internal MF
aiMT: anterior internal MT
HTO: high tibial osteotomy
mcMF: middle central MF
mcMT: middle central MT
meMF: middle external MF
meMT: middle external MT
MF: medial femur
MFC: medial femoral condyle
miMF: middle internal MF
miMT: middle internal MT
MMCR: medial meniscus covering ratio
MME: medial meniscus extrusion
MT: medial tibia
MTP: medial tibial plateau
OA: osteoarthritis
pcMF: posterior central MF
pcMT: posterior central MT
peMF: posterior external MF
peMT: posterior external MT
piMF: posterior internal MF
piMT: posterior internal MT
ROI: region of interest

## INTRODUCTION

Medial meniscus extrusion (MME) leads to reduced coverage of the tibial plateau, causing the development and progression of knee osteoarthritis (OA) [19, 20, 29]. MME was found to be associated with progression of cartilage structure destruction and incidence of knee arthroplasty independent of age, gender, and body mass index in a systematic review [6], indicating that cartilage coverage by the meniscus is important for the long‐term maintenance of healthy cartilage. In addition, MM posterior root tear (MMPRT) causes severe MME, sharply increases the risk of knee OA, and increases the risk of total knee arthroplasty [14]. Degenerative meniscus tears, which are generally seen in the elderly, also progress slowly but do not significantly affect the prognosis of OA, and meniscus extrusion is considered a more important factor in knee OA [18].

Three‐dimensional (3D) magnetic resonance imaging (MRI) analysis has been developed to clarify the pathology of knee OA [2, 21]. In a 12‐month follow‐up study, knees progressively losing cartilage, as evaluated by 3D MRI, showed significantly greater MME obtained from the central slice of the meniscus compared with knees without cartilage loss progression [26]. However, concerning the morphology of the meniscus, only height impacted the progression of cartilage loss; width or volume had no influence. Additionally, the meniscal coverage of the tibial plateau was unaffected. The limitations of the previous 3D MRI analysis include the manual segmentation used to delineate the cartilage and meniscus. This manual process introduced subjectivity into the quantitative analysis of both the cartilage and the meniscus.

The advancement of deep learning has further improved 3D analysis in MRI and has enabled the automatic segmentation and reconstruction of MRI data [8]. This system can not only map cartilage thickness [1] but also determine the average cartilage thickness and volume for each subregion in approximately 5 min [25]. The average cartilage thickness correlated with arthroscopic cartilage grade in 11 of the 15 subregions in the medial femoral and tibial regions [17]. This system also allows for the evaluation of the medial meniscal coverage ratio (MMCR), which is defined as the ratio of the area covered by the meniscus within the medial tibial cartilage area to the total medial tibial cartilage area [1]. In healthy knee joints, MMCR was significantly correlated with the cartilage area ratio at the middle center of the medial tibial plateau, where the ratio represents the proportion of the cartilage area in the region of interest (ROI) and where the cartilage is present at a thickness greater than a certain threshold [1]. However, the correlation between MMCR and cartilage thickness in patients with knee OA has not been clearly established. The purpose of this study was to retrospectively investigate whether average cartilage thickness calculated by an automatic 3D MRI analysis system is correlated with MMCR. Our hypothesis is that cartilage loss assessed by MRI 3D analysis would correlate with MMCR and that the strength of this correlation would vary by subregion.

## METHODS

### Subjects

This study was approved by the internal review board of our institution, and informed consent was obtained from all patients. The study included 102 patients who underwent arthroscopy prior to undergoing isolated meniscus surgeries and high tibial osteotomies (HTO). Patients who had lateral meniscus surgery were excluded from the sample. Finally, 60 patients who had isolated medial meniscus surgeries (*n* = 23) and HTO for medial OA (*n* = 37) were included. Kellgren–Lawrence (KL) grade was evaluated by x‐ray.

### KL grading

Radiographs were taken of the anteroposterior view of the knee with the patient in a standing position with full knee extension [16], and KL grade was evaluated as previously reported [7].

### MRI scanning

MRI was performed at 3.0 T (Achieva 3.0TX; Philips) with 16‐channel coils. Sagittal planes of the knee joints were acquired to obtain both fat‐suppressed spoiled gradient echo sequence images and proton‐weighted images, with total scan durations of 7 min 30 s and 7 min 10 s, respectively. Both types of sagittal images were obtained at an in‐plane resolution of 0.31 × 0.31 mm, a partition thickness of 0.36 mm (320 slices), and a field of view (head to tail × anterior to posterior) of 150 × 150 mm.

### Measurements of cartilage thickness using 3D MRI

**Figure 1:**
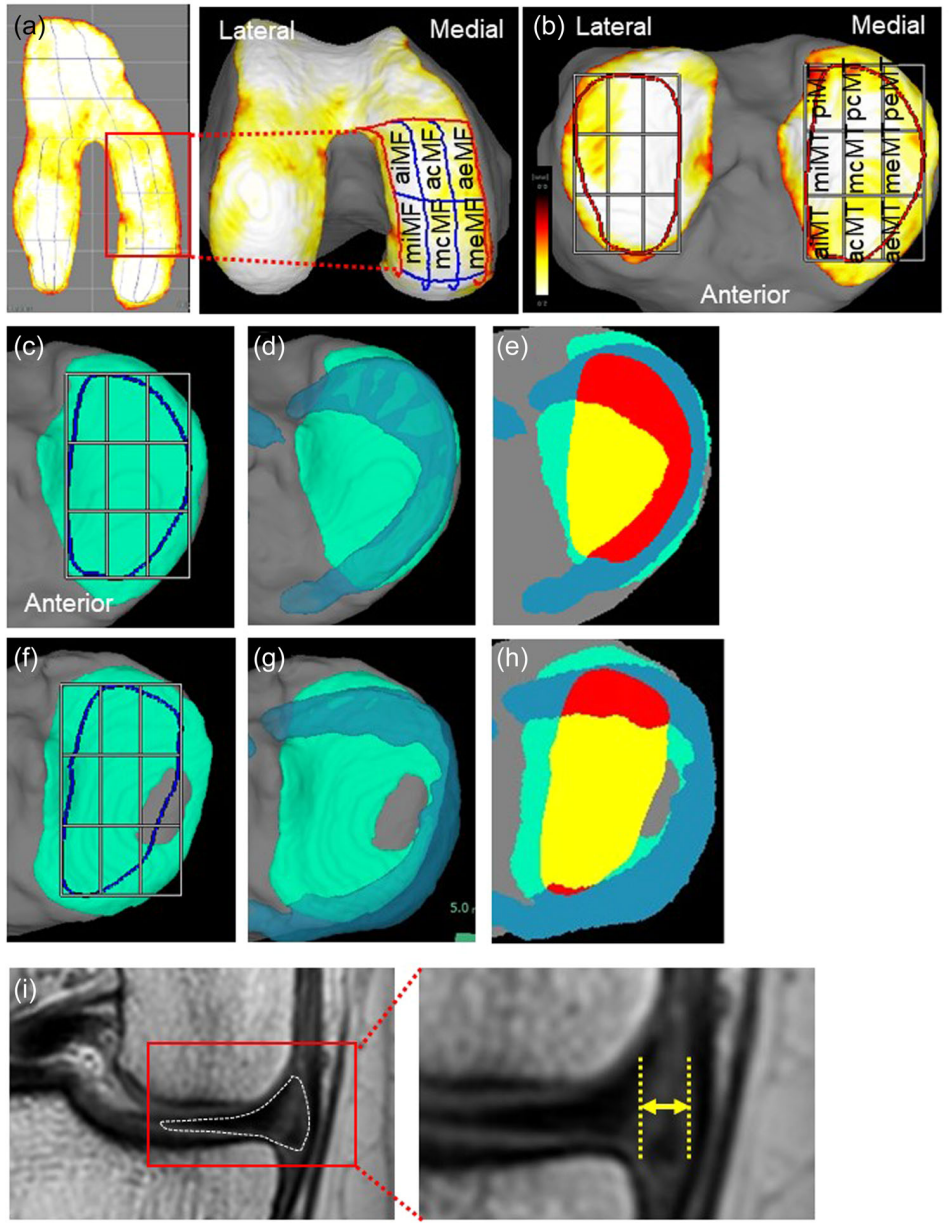
Analysis of the medial meniscus and cartilage of the medial femoral condyle and the medial tibial plateau in three‐dimensional (3D) and two‐dimensional (2D) magnetic resonance imaging (MRI). (a) Radially (left) and vertically (right) projected cartilage thickness mapping and subregions of the medial femoral cartilage. acMF, anterior central MF; aeMF, anterior external MF; aiMF, anterior internal MF; MF, medial femur; mcMF, middle central MF; meMF, middle external MF; miMF, middle internal MF. (b) Vertically projected cartilage thickness mapping and subregions of the tibial cartilage. acMT, anterior central MT; aeMT, anterior external MT; aiMT, anterior internal MT; mcMT, middle central MT; meMT, middle external MT; miMT, middle internal MT; MT, medial tibia; pcMT, posterior central MT; peMT, posterior external MT; piMT, posterior internal MT. (c) Medial compartment of the tibia in a healthy knee. The cartilage is coloured in green, and the region of interest (ROI) for the cartilage is represented in blue lines. (d) Medial tibial cartilage with the medial meniscus (MM) in a healthy knee. MM is shown in light blue. (e) Medial meniscus covering ratio (MMCR) in a healthy knee. The portion of the MM within the cartilage ROI is indicated in red, while the portion outside the ROI is shown in blue. The area of the cartilage not covered by the MM within the ROI is marked in yellow. MMCR refers to the ratio of the red area to the sum of the yellow and red areas. (f) The medial compartment of the tibia in a case involving medial meniscus extrusion (MME). The tibial cartilage is indicated in green, while the ROI is shown in blue lines. (g) Medial tibial cartilage and the MM in a case of MME. MM is shown in light blue. (h) MMCR in the case of MME. (i) MME width analysed by 2D MRI coronal plane. The left panel shows the medial compartment of the knee. The right panel shows a high‐magnification image of the MM. The white dotted line represents the MM, and the yellow arrow indicates MME width.

The MRI DICOM data were read by the SYNAPSE VINCENT 3D software (Japanese product name: SYNAPSE VINCENT), collaborative version, FUJIFILM Corporation). The bone and cartilage regions were automatically extracted, and the femoral cartilage was projected radially around the intercondylar axis, which connected the centres of the medial and lateral condyles. The software mapped cartilage thickness by displaying cartilage thickness as a colour scale, with the thick areas of cartilage indicated in white and the thin areas in red. The software automatically drew a closed curve line for the ROI based on the bone contour; then, it divided the ROI into three regions based on the shape of the ROI, and then further divided each region into nine subregions based on the shape of each region. The software also showed the 3D femoral cartilage viewed from below along the long axis of the femur (Figure 1a). The Dice similarity coefficient for automatic segmentation accuracy was 0.911 for femoral cartilage and 0.892 for tibial cartilage [1], and the interscan measurement error of the knee cartilage thickness for the 18 subregions of the medial femoral region and the medial tibial region ranged from 0.03 mm to 0.11 mm [24]. In the medial tibial region, the software automatically drew closed curve lines for the ROI based on the bone contour. The ROI was divided into nine subregions with three equally sized vertical and horizontal divisions (Figure 1b). In the present study, only the cartilage thickness of the medial compartment was analysed. A previous study validated the use of 3D MRI in arthroscopy and found that the posterior regions of the femoral cartilage shown by 3D MRI were not assessed by arthroscopy [17]. The region was referred to as the hidden zone; [22] therefore, the analysis of the femoral cartilage was limited to the anterior and middle subregions and excluded the posterior subregions (Figure 1a). Lastly, the medial tibial plateau was evaluated at nine subregions (Figure 1b).

### Measuring meniscus covering ratio with 3D MRI

The medial meniscus covering ratio (MMCR) was defined as the ratio of the overlapping area of the medial meniscus and the area of ROI of the medial tibial plateau [1]. The area of the cartilage is indicated in green (Figure 1c,d), and the ROI for the assessed cartilage is marked by a blue line (Figure 1d). The portion of the meniscus within the cartilage ROI is indicated in red, while the portion outside the ROI is depicted in blue (Figure 1e). Cartilage not covered by the meniscus within the ROI is marked in yellow. MMCR is calculated as the ratio of the red area and the sum of the yellow and red areas (Figure 1e).

### Measurement of MME using 2D MRI

MME width was retrospectively measured by one orthopaedic surgeon (N.O.) who had more than 20 years of experience in this field. MME width was measured from the most peripheral aspect of the meniscus to the border of the tibia, excluding osteophytes on the mid‐coronal slice, in proton density‐weighted images (Figure 1f) [28]. Intra‐observer measurement reproducibility was assessed with the intraclass correlation coefficient (ICC). The observer was blinded to the previous results, and the intra‐observer ICC was 0.97.

### Statistical analysis

Spearman's rank correlation coefficient (*rs*) was used to evaluate the association between the ICRS grade and the average cartilage thickness at each subregion. For cartilage thickness, a *p*‐value of *p* < 0.05 was considered statistically significant. JMP14® (SAS Institute Inc.) was used for all statistical analyses. Correlation coefficients of 0.00–0.10 were considered “negligible,” 0.10–0.39 as “weak,” 0.40–0.69 as “moderate,” 0.70–0.89 as “strong,” and 0.90–1.00 as “very strong” [23]. “A power analysis was performed with statistical software (G*Power, v 3.1.9.7; Heinrich Heine Universität Düsseldorf) before the investigation. A sample size of 42 patients was needed for an *α* of 0.05, 1 − *β* of 0.95, an effect size of 0.5, and a power of 0.95.”

## RESULTS

### Patient characteristics: 2D and 3D MRI data

Patient characteristics included a total of 60 subjects whose ages ranged from 14–74 years of age, with a median age of 57 years (Table 1). There were 31 male subjects and 29 female subjects. In terms of laterality, 35 cases were related to the right knee, and 25 to the left knee. The diagnoses included 23 knees with meniscus injuries and 37 knees with OA. The distribution of KL grades showed six knees at Grade 0, eight at Grade 1, 10 at Grade 2, 25 at Grade 3, and 11 at Grade 4. The average MMCR was 0.47, while the average MME width in the 2D MRI was 3.6 mm (Table 2). Median cartilage thickness at subregions evaluated by 3D MRI was presented in Table 3.

**Table 1:** Patient characteristics (*n* = 60).

Age (median, range)	57 (14–74)
Sex	
Male	31
Female	29
Laterality	
Right	35
Left	25
Diagnosis	
Meniscus injury	23
Osteoarthritis	37
Kellgren–Lawrence (KL) grade	
0	6
1	8
2	10
3	25
4	11

**Table 2:** Meniscus coverage ratio and medial meniscus extrusion (*n* = 60).

	Median	Average	Std
MMCR	0.47	0.45	0.16
MME	3.6	3.7	2.1

Abbreviations: MMCR, medial meniscus covering ratio; MME, medial meniscus extrusion; Std, standard deviation.

**Table 3:** Median cartilage thickness at subregions evaluated by 3D MRI (*n* = 60).

Medial femoral condyle (mm)			Medial tibial plateau (mm)		
	Median	Range		Median	Range
meMF	1.6	(1.5–3.2)	peMT	1.5	(0–2.6)
aeMF	1.4	(0–2.5)	meMT	1.3	(0–2.1)
mcMF	1.8	(0.2–2.7)	aeMT	1.5	(0–2.7)
acMF	1.6	(0–2.6)	pcMT	1.8	(0.6–2.8)
miMF	1.9	(0.6–2.7)	mcMT	1.8	(0–2.9)
aiMF	1.9	(0.2–2.8)	acMT	1.7	(0–2.4)
			piMT	1.8	(1.2–2.8)
			miMT	2.5	(1.4–3.7)
			aiMT	1.8	(0.1–3.0)

Abbreviations: 3D MRI, three‐dimensional magnetic resonance imaging; acMF, anterior central MF; acMT, anterior central MT; aeMF, anterior external MF; aeMT, anterior external MT; aiMF, anterior internal MF; aiMT, anterior internal MT; MF, medial femur; mcMF, middle central MF; mcMT, middle central MT; meMF, middle external MF; meMT, middle external MT; miMF, middle internal MF; miMT, middle internal MT; MT, medial tibia; pcMT, posterior central MT; peMT, posterior external MT; piMT, posterior internal MT.

### Correlations among KL grade, MMCR, and MME width

**Figure 2:**
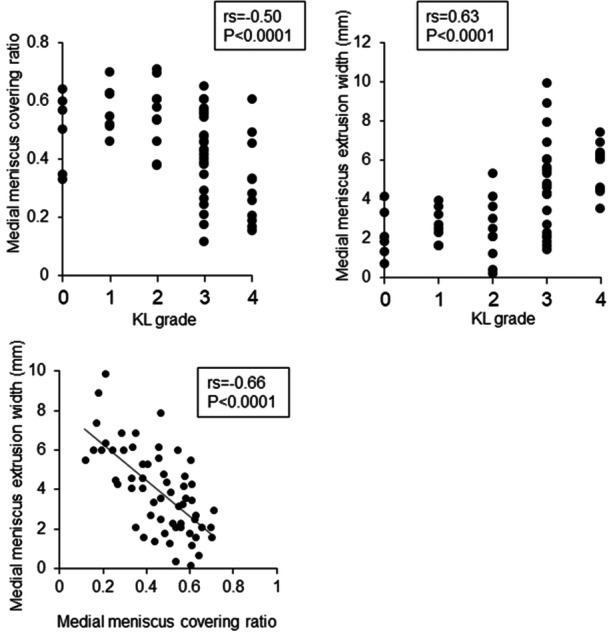
Correlation between KL grade and medial meniscus covering ratio, KL grade and medial meniscus extrusion width, and MMCR and MME width. Spearman's rank correlation coefficient (*rs*) and *p*‐values are also shown (*n* = 60). KL, Kellgren–Lawrence; MMCR, medial meniscus covering ratio; MME, medial meniscus extrusion.

KL grade had a negative correlation with MMCR (*rs* = –0.50) and a positive correlation with MME width (*rs* = 0.63) (Figure 2). MMCR had a significantly negative correlation with MME width (*rs* = 0.66).

### Correlations between cartilage thickness and MMCR

**Figure 3:**
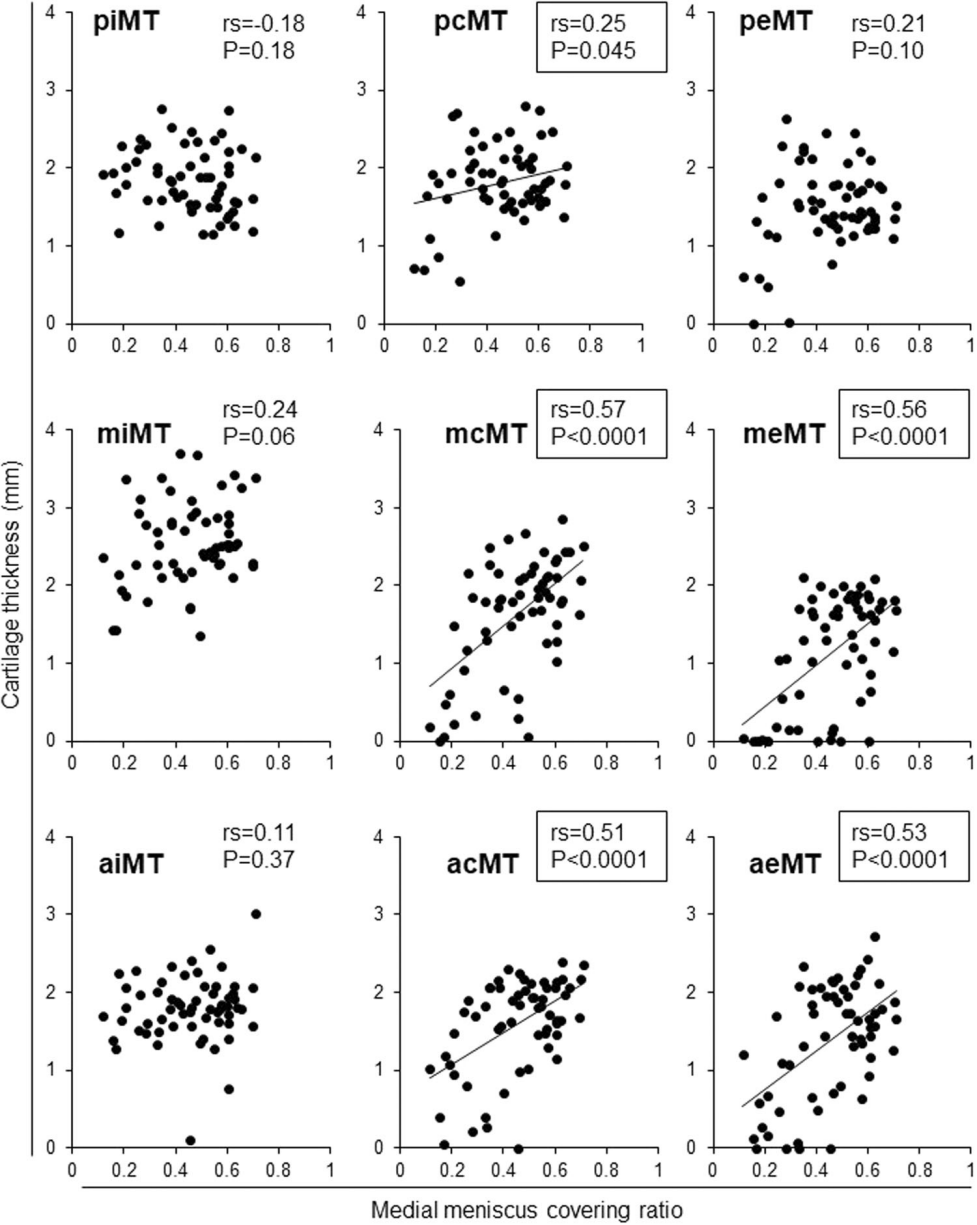
Correlation between the medial meniscus covering ratio and cartilage thickness in nine medial tibial subregions. Spearman's rank correlation coefficient (*rs*) and *p*‐values are also shown (*n* = 60). Subregions with *p* < 0.05 are enclosed in squares. acMT, anterior central MT; aeMT, anterior external MT; aiMT, anterior internal MT; mcMT, middle central MT; meMT, middle external MT; miMT, middle internal MT; MT, medial tibia; pcMT, posterior central MT; peMT, posterior external MT; piMT, posterior internal MT.

**Figure 4:**
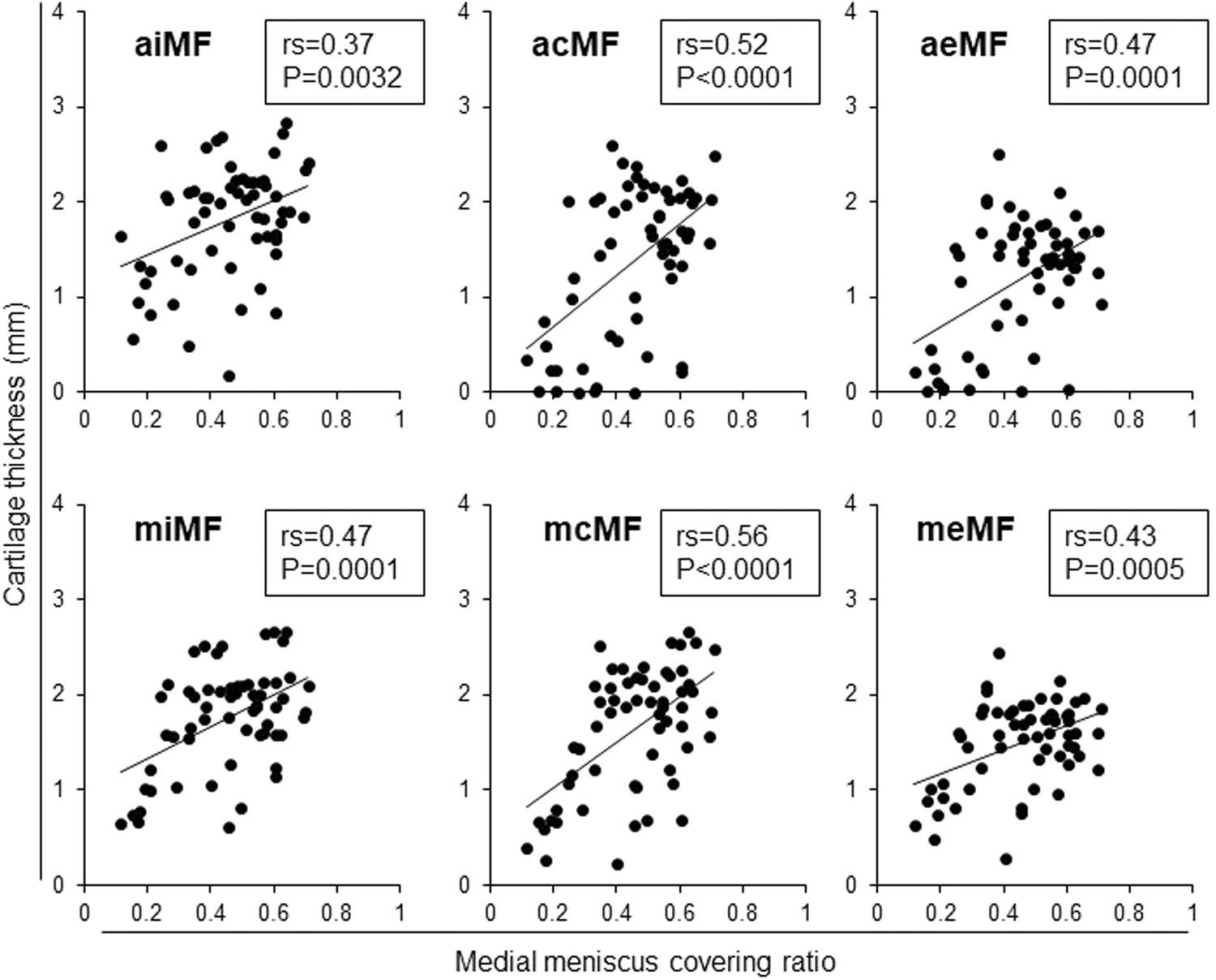
Correlation between the medial meniscus covering ratio and cartilage thickness in six medial femoral subregions. Spearman's rank correlation coefficient (*rs*) and *p*‐values are also shown (*n* = 60). Subregions with *p* < 0.05 are enclosed in squares. acMF, anterior central MF; aeMF, anterior external MF; aiMF, anterior internal MF; MF, medial femur; mcMF, middle central MF; meMF, middle external MF; miMF, middle internal MF.

Cartilage thickness in the medial tibial subregions had a moderately positive correlation with MMCR at the acMT, aeMT, mcMT, and meMT subregions. It had a weakly positive correlation at the pcMT subregion (Figure 3). Cartilage thickness in the medial femoral subregions showed a moderately positive correlation with MMCR at the acMF, aeMF, miMF, mcMF, and meMF subregions and a weakly positive correlation at the aiMF subregion (Figure 4).

### Correlations between cartilage thickness and MME width

**Figure 5:**
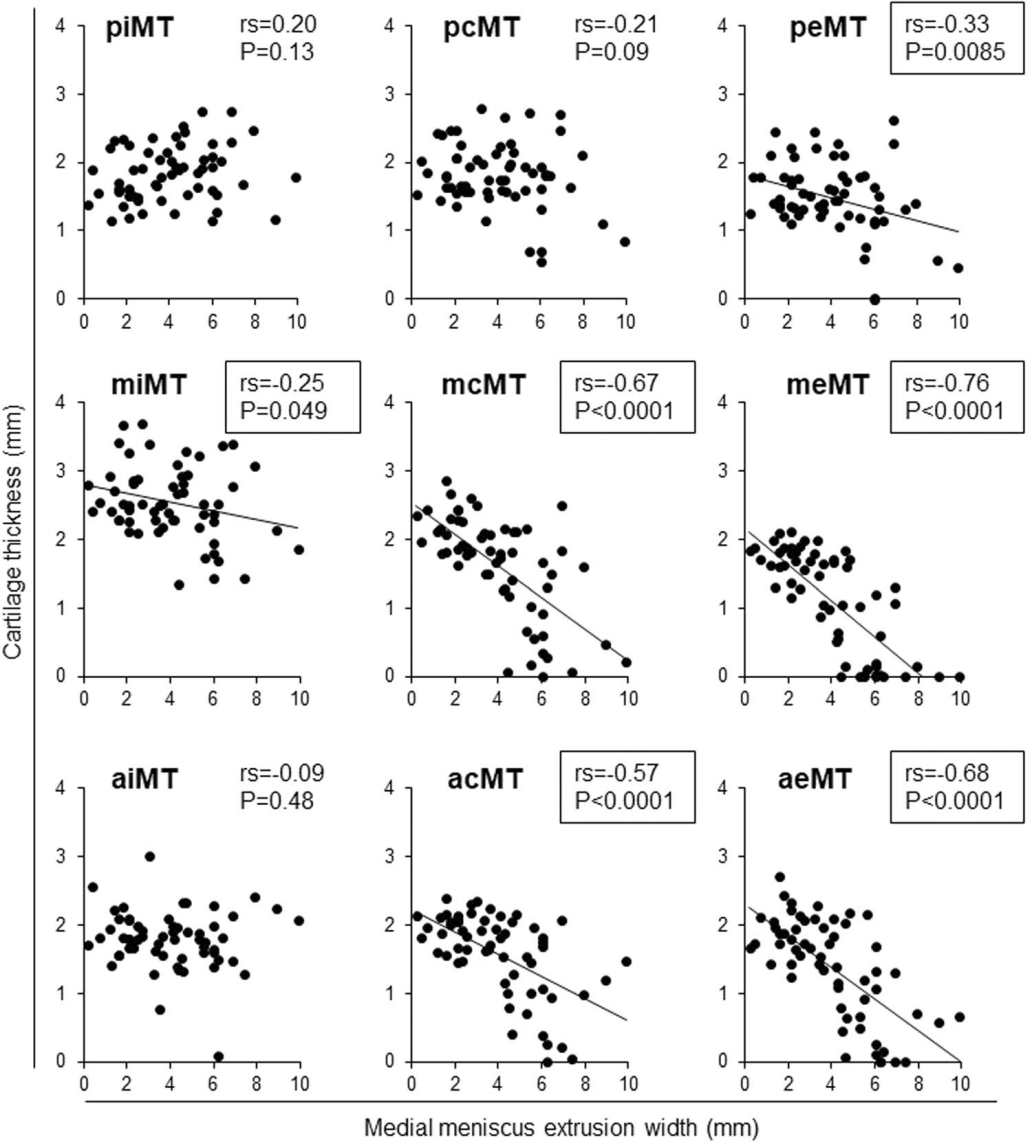
Correlation between medial meniscus extrusion width and average cartilage thickness in nine medial tibial subregions. Spearman's rank correlation coefficient (*rs*) and *p*‐values are also shown (*n* = 60). Subregions with *p* < 0.05 are enclosed in squares. acMT, anterior central MT; aeMT, anterior external MT; aiMT, anterior internal MT; mcMT, middle central MT; meMT, middle external MT; miMT, middle internal MT; MT, medial tibia; pcMT, posterior central MT; peMT, posterior external MT; piMT, posterior internal MT.

**Figure 6:**
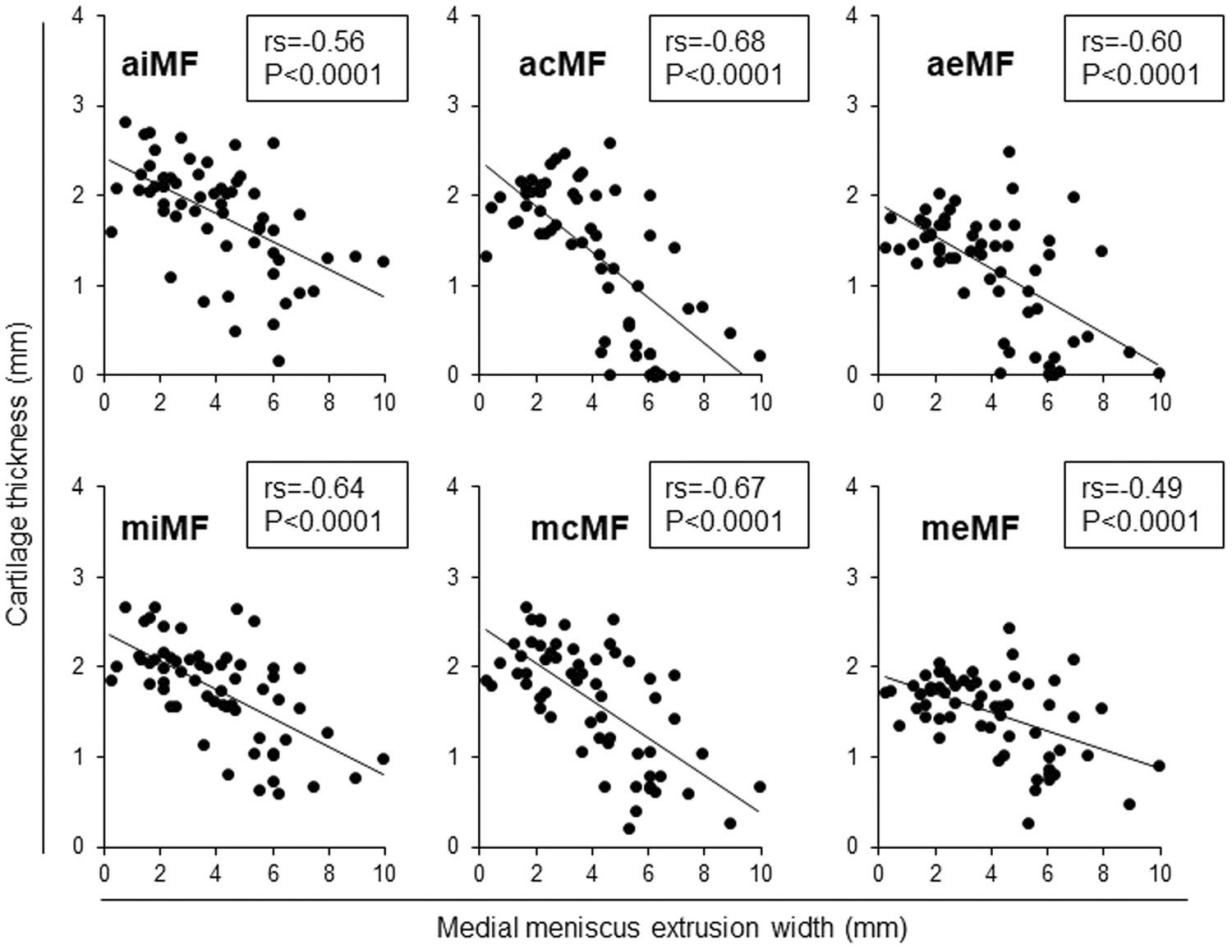
Correlation between the medial meniscus extrusion width and cartilage thickness in six medial femoral subregions. Spearman's rank correlation coefficient (*rs*) and *p*‐values are also shown (*n* = 60). Subregions with *p* < 0.05 are enclosed in squares. acMF, anterior central MF; aeMF, anterior external MF; aiMF, anterior internal MF; MF, medial femur; mcMF, middle central MF; meMF, middle external MF; miMF, middle internal MF.

Cartilage thickness in the medial tibial subregions was moderately negatively correlated with MME width at the acMT, aeMT, mcMT, and meMT subregions and weakly negatively correlated at the miMT and peMT subregions (Figure 5). Cartilage thickness in the medial femoral subregions was negatively correlated with MME width at the aiMF, acMF, aeMF, miMF, mcMF, and meMF (Figure 6).

## DISCUSSION

We retrospectively investigated whether the average cartilage thicknesses calculated for 15 subregions in the medial femoral and tibial cartilage were correlated with the MMCR using a 3D MRI analysis system. The most important finding was that cartilage thicknesses in the MTP by 3D MRI were positively correlated with MMCR for four regions of six subregions of the anterior and middle subregions in the MTP. To the best of our knowledge, this is the first study to investigate the correlation between cartilage thickness and MMCR calculated by automatic 3D MRI software.

MMCR and MME width correlated with KL grade, the most common classification of OA severity. This result is similar to that of a previous study that showed that meniscal extrusion is highly associated with symptomatic knee OA [5]. However, the KL grade is incapable of discerning the specific location of cartilage loss, whether on the femoral or tibial side, anterior or posterior regions, or central or peripheral regions. A previous study indicated that joint space narrowing in the Rosenberg view correlated with a higher volume of MME, yet the study failed to pinpoint the precise location of cartilage loss [16]. In our study, we not only assessed the KL grade but also examined cartilage thickness in each subregion. This approach allowed us to identify the specific regions most affected by cartilage loss.

The MMCR exhibited a moderate and negative correlation with MME width. Typically, the MME measurement is carried out on one or several slices in the coronal plane of 2D MRI [3, 9], a procedure similar to ultrasound [11, 27]. However, as the shape of the meniscus is circular, relying solely on evaluations in the coronal plane may not fully capture the pathology of MME. Moreover, MME tends to be larger in individuals of larger body size and smaller in those with smaller bodies. In addition, such evaluations are typically measured by physicians and can thus be subjective. In contrast, MMCR is a quantitative ratio that considers cartilage covered and uncovered by the meniscus within the ROI. Importantly, it is not influenced by body size. Additionally, in this study, the cartilage and meniscus were automatically extracted, and the ratio was calculated objectively by 3D MRI software—a completely objective scoring system for assessing meniscal coverage of the cartilage.

Our study revealed a positive correlation between cartilage thickness and MMCR in five out of nine subregions of the MTP and all subregions of the MFC. A previous 3D MRI analysis revealed that the size of the MM uncovered by the joint surface was significantly larger in knees with OA than in non‐OA knees, with both the mean and MME being significantly greater in OA knees [30]. In addition, cartilage defects originated in the middle external subregion, extending seamlessly into the surrounding area without dissociation from the inner margin of the meniscus, concurrent with typical meniscus extrusion [10]. These results showed that the coverage of the meniscus is critical for cartilage preservation. Furthermore, our results demonstrated a negative correlation between cartilage thickness and MME width in six out of nine subregions of the MTP and all subregions of the MFC. A biochemical study demonstrated that load distribution in the intact knee is mainly over the entire meniscus, shifting the concentration of the tibial plateau with slight distribution on the meniscus during meniscus extrusion [15]. MME width is closely linked to the development of knee OA and is attributed to the reduced coverage of the meniscus. Recent advancements in surgical techniques, particularly the centralisation of the extruded meniscus [4, 12, 13], which were not previously performed for conditions involving meniscal extrusion, can now possibly preserve meniscus function by increasing meniscus coverage.

**Figure 7:**
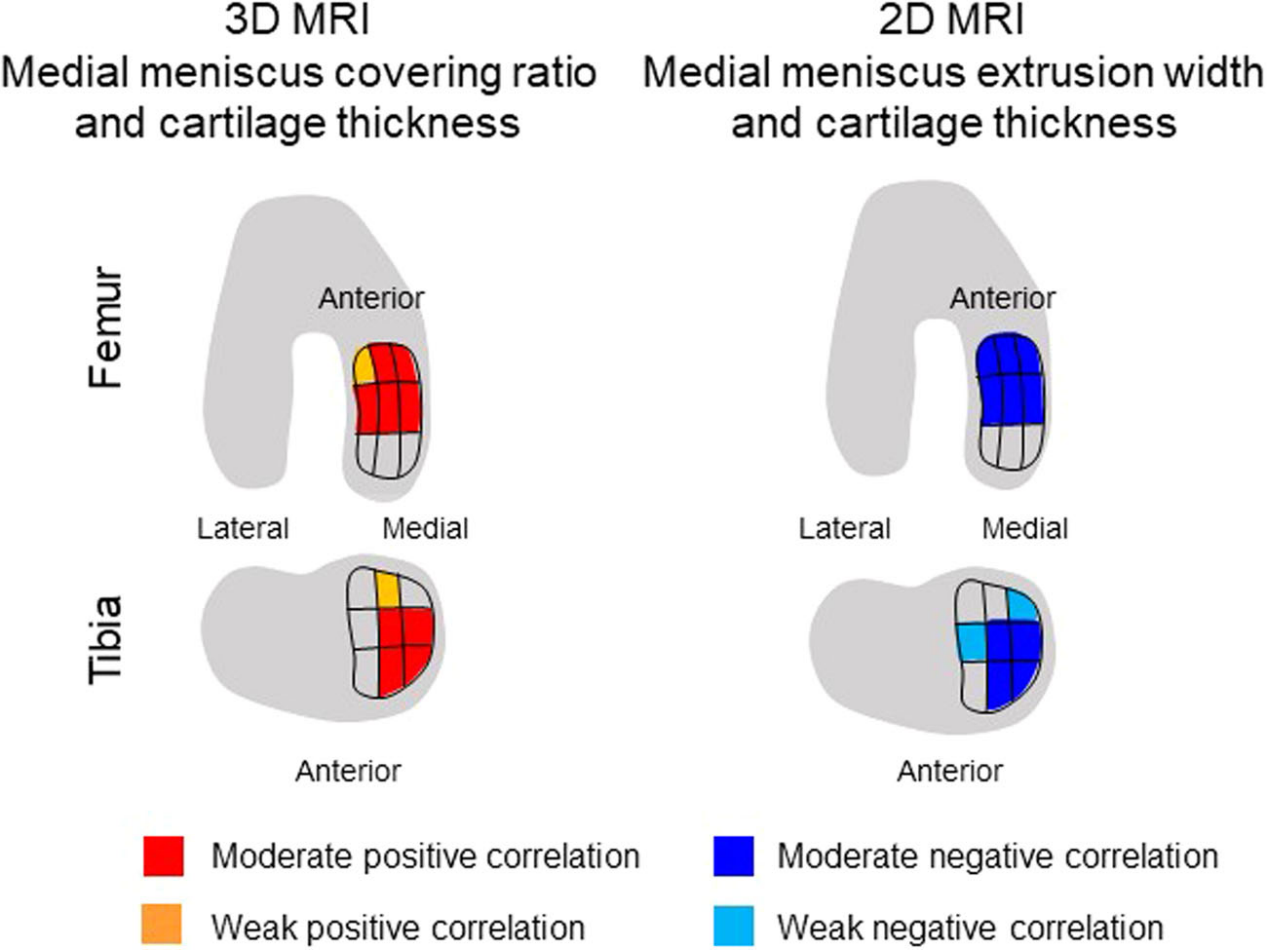
Summary of the correlation between medial meniscus covering ratio and cartilage thickness and between medial meniscus extrusion width and the thickness in 10 medial compartment subregions. The red area indicates a moderate positive correlation, the orange area depicts a weak positive correlation, the blue area shows a moderate negative correlation, and the light blue area details a weak negative correlation. Cartilage loss was observed in the anterior and medial portions of the femoral condyles and in the anterior and medial portions of the central and external portions of the medial tibial plateau in a knee with decreased MMCR due to an increased MME width. Conversely, the cartilage remained relatively preserved in the posterior subregions of the medial tibial plateau. 2D MRI, two‐dimensional magnetic resonance imaging; 3D MRI, three‐dimensional magnetic resonance imaging; MMCR, medial meniscus covering ratio; MME, medial meniscus extrusion.

Based on our findings, we have summarised the association between reduced cartilage thickness and MMCR and the relationship between reduced cartilage thickness and MME width in knee OA (Figure 7). In a knee with a decreased MMCR and an increased MME width cartilage loss was observed in the anterior and medial portions of the femoral condyles and in the anterior and medial portions of the central and external portions of the MTP. Conversely, the cartilage remains relatively preserved in the posterior subregions of the MTP. This is caused by the maintained meniscal coverage in these areas. Further studies are necessary to clarify the relationship between the location of cartilage loss and the location of the MME.

Our current study has two limitations. First, as this is a retrospective study, we did not assess the progression of cartilage loss or MMCR reduction over time. A prospective study to investigate whether decreased MMCR impacts cartilage thickness, as assessed by 3D MRI, is necessary to achieve a more comprehensive understanding. Second, our evaluation was limited to the medial compartment, as we specifically chose patients who underwent surgeries related to the medial meniscus or HTO. Consequently, our patient cohort exhibited minimal cartilage lesions in the lateral compartment. An analysis of the lateral meniscus covering ratio with cartilage thickness in the lateral compartment is essential to elucidate the pathology of lateral knee OA.

In conclusion, the cartilage thickness calculated by the automatic MRI 3D analysis system had a positive correlation with MMCR for all subregions of the anterior, all middle subregions in the MFC, and five out of nine subregions of the anterior and middle regions in the MTP.

## AUTHOR CONTRIBUTIONS

Nobutake Ozeki contributed to the study's conception and design, collection of data, analysis and interpretation of data, and manuscript writing. N.K., Hideyuki Koga, Yusuke Nakagawa, Takashi Hoshino, Mai Katakura, Masaki Amemiya, and Aritoshi Yoshihara contributed to the collection and interpretation of the data. Mitsuru Mizuno, Hisako Katano, Kentaro Endo, Jun Masumoto, and Hideyuki Koga contributed to the interpretation of the data. Ichiro Sekiya contributed to the study's conception and design, financial support, manuscript writing, and final approval of the manuscript.

## CONFLICT OF INTEREST STATEMENT

Ichiro Sekiya has funding from FUJIFILM Corporation. The authors declare no conflicts of interest.

## ETHICS STATEMENT

This study was approved by the Medical Ethics Committee of Tokyo Medical and Dental University, and informed consent was obtained from all patients. Research Protocol Identification Number: M2023‐088.

## Data Availability

The data that support the findings of this study are not publicly available.
